# *In-situ* magnetization/heating electron holography to study the magnetic ordering in arrays of nickel metallic nanowires

**DOI:** 10.1063/1.5007671

**Published:** 2018-01-09

**Authors:** Eduardo Ortega, Ulises Santiago, Jason G. Giuliani, Carlos Monton, Arturo Ponce

**Affiliations:** 1 Physics and Astronomy, University of Texas at San Antonio, San Antonio, TX, 78249, United States; 2 National Institute of Astrophysics, Optics and Electronics, INAOE, Tonantzintla, Puebla 72840, Mexico

## Abstract

Magnetic nanostructures of different size, shape, and composition possess a great potential to improve current technologies like data storage and electromagnetic sensing. In thin ferromagnetic nanowires, their magnetization behavior is dominated by the competition between magnetocrystalline anisotropy (related to the crystalline structure) and shape anisotropy. In this way electron diffraction methods like precession electron diffraction (PED) can be used to link the magnetic behavior observed by Electron Holography (EH) with its crystallinity. Using off-axis electron holography under Lorentz conditions, we can experimentally determine the magnetization distribution over neighboring nanostructures and their diamagnetic matrix. In the case of a single row of nickel nanowires within the alumina template, the thin TEM samples showed a dominant antiferromagnetic arrangement demonstrating long-range magnetostatic interactions playing a major role.

High-density arrays of magnetic nanowires (NWs) have attracted significant interest due to their expected technological applications in perpendicular recording media,^1^ magnetoelectronic devices,^2^ sensors,^3,4^ and power devices.^5^ Research is being realized to design dense assemblies of nanomagnets where each bit comprises a single magnetic object non-volatile and easy to read/modify,^6^ as traditional storage devices have intrinsic limitations around 1 Tbits/in^2^.^7^ Typical methods to prepare closely packed nanowires rely on the use of low cost nanoporous membranes which results in polycrystalline structures arranged into templates.^8^ Understanding of the effective interaction fields on the intrawire and interwire regime and their effect on the macroscopic response is essential if we wish to control the switching fields to use these structures as independent memory bits. Usually, the magnetic measurements of the complete array are performed using vibrating sample magnetometers (VSM),^9,10^ but only a few studies have been done to analyze the local response of the nanowires-anodic aluminum oxide (AAO) composite.^11–13^

Advanced surface techniques such as photoemission electron microscopy (PEEM),^14^ magnetic force microscopy (MFM),^15^ Lorentz microscopy^16^ and electron holography (EH)^17^ can be used to locally evaluate the magnetic interactions of individual cylindrical nanowires and arrays.^18^ As an example, the work by Kovács et al.^19^ over a Fe-Si-B-Nb-Cu alloy focused ion beam (FIB) milled lamellae was able to highlight a regular domain pattern over a stress-annealed sample and recently Zu et al.^20^ found a correlation between the AlNiCo microstructures and their reversal mechanism on an array of two different AlNiCo phases (needles and matrix), showing the importance of nanoscale measure techniques to study and understand fine local interactions.

In this work, we characterized the microstructure of electrodeposited Nickel nanowires using transmission electron microscopy (TEM) related techniques. Combing EH, VSM and micromagnetic simulations, we show the magnetic ordering and magnetic field distribution of ferromagnetic Ni inside the template after using FIB to acquire a single row of uniformly spaced nanowires.

The nickel nanowires were synthesized, by electrochemical deposition using an AAO porous membrane as a template. The AAO was fabricated using the two-step anodization process.^8^ The resulting AAO had had a 700 nm pore depth and 80 nm pore diameter and was transferred to a silicon substrate (Fig. 1a), coated with a 150 nm silver layer, which was used as the working electrode during the chronopotentiometric electrodeposition process. The electrodeposition was performed in a commercial solution of nickel sulfamate RTU (Technic Inc.) at a constant current of 1.37 mA. The total electrodeposition time was 5.9 minutes, which resulted in the nickel overgrowing in some regions of the AAO pores.

**FIG. 1.**
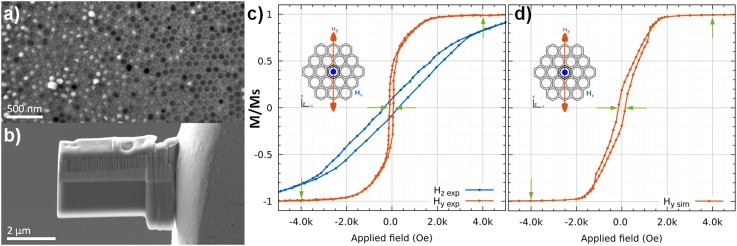
(a) Plain view SEM images for the as-prepared hexagonal packed Ni/AAO membrane precursor, (b) FIB lamella during 30 KV Ga+ milling, (c) Magnetic hysteresis curves of Ni nanowires measured inside the membrane under an external field parallel (Hz-blue) and perpendicular (Hy-orange) to the nanowire long axis, (d) Simulated magnetic response over the nanowire long axis, coercivity and saturation points are highlighted.

Array magnetic response was analyzed by vibrating sample magnetometer (VSM, Lakeshore 7304). Samples were prepared for TEM analysis using a Zeiss Crossbeam 340 FIB working with 30, 5 and 2kV gallium ions (Fig. 1b). The TEM work was performed in a JEOL ARM 200F, operated at 200 kV. To determine the crystallinity of the nanowires two diffraction techniques were involved: selected area electron diffraction (SAED) and precession electron diffraction-assisted automated crystal orientation mapping (PED ACOM-TEM) using Nanomegas hardware and software package.^21^

To perform PED, the system works under nanobeam electron diffraction conditions, using the smallest condenser aperture and a probe size of approximately 1.5 nm in diameter. The electron beam is scanned across the sample and collects the electron diffraction patterns using an external CCD camera recording the viewing screen of the microscope. An electron diffraction pattern (DP) is collected by pixel and then indexed using an automatic matching procedure, which correlates the data with preselected theoretical templates from a database. The use of PED allows enhancing the quality of the recorded DPs since PED reduces the dynamic effects and increase the intensity of the diffraction spots.^22^ All off-axis electron holography data acquisition was acquired under Lorentz conditions. In situ heating was performed with a heating holder model EM-21130.

The room temperature magnetic response of the full composite is shown in Fig. 1c with magnetic Field (H) applied in two directions, i.e., parallel and perpendicular to the wire direction. From the hysteresis loops, we can observe a strong shape anisotropy; the magnetocrystalline anisotropy can be neglected due to the strong inter-grain exchange coupling in each nanowire.^24^ The sample seems to prefer to be magnetized (easy axis) when the field is applied perpendicular to the NWs. This is a result of two contributions; the Ni layer that partially covers the NWs at the top of the sample, and the high density of NWs in the plane of the sample. Both, plays a strong role in modifying the shape anisotropy of the system. Consequently, the shape of the magnetization loops is the result of the combined effects of the distribution of the NWs and the top Ni film. To corroborate the magnetic response obtained with VSM the hysteresis loop curves of the configuration was simulated and their results shown in Fig 1d (using Nmag software package^23^). In hysteresis calculation it was considered the influence over the central element done by 120 outer elements (within a hexagonal array). To plot the data, a magnetic field from 2.4×10^6^ A/m (∼3×10^4^ Oe) to -2.4×10^6^ A/m was applied in armchair direction (H_y_) as applied inside the TEM (inset in Fig 1d). Both curves show similar behavior in coercivity and saturation values.

Structural analysis was achieved using selected area electron diffraction (SAED) to analyze the crystallinity of the array prepared by FIB. Electron diffraction patterns were captured in a 600 nm diameter field of view enclosing evenly spaced neighboring nanowires that share the same direction as shown in the TEM micrograph of Fig. 2a. The inset electron diffraction pattern shows a large selection of reflections with its closest diffraction rings at 0.197, 0.170 and 0.121 nm corresponding to the distance between (111), (200) and (220) planes of the Ni FCC structure. From the SAED a texturized pattern was not appreciable, the homogeneous distribution of the ring intensities suggests a collection of randomly oriented crystals during the electrodeposition process. To coroborate the previous results and to visualize the grain size, distribution and misorientatons Fig. 2b displays the crystal orientation map of the same region acquired and indexed by a PED unit. The crystal orientation map appears concerning the “z” direction of the image plane, which is the direction perpendicular to the lamella. All crystal orientation maps have been overlapped with the index map (which measures the degree of correlation) to improve data representation.^21^ The inset on Fig 2b gives the color map code to interpret the grains orientationd. As expected, the nanowires revealed a polycrystalline nature, comprising a variety of grain sizes and orientations There is a tendency of having small grains with variable orientations towards the end of NWs; whereas larger grains can be noticed towards the base. Using the Nanomegas software, once the full orientation map is indexed, the misorientation angle between neighboring crystals can be obtained, an histogram of these values shows a collection of grains misoriented between 18° and 60° with a median of 42°.

**FIG. 2.**
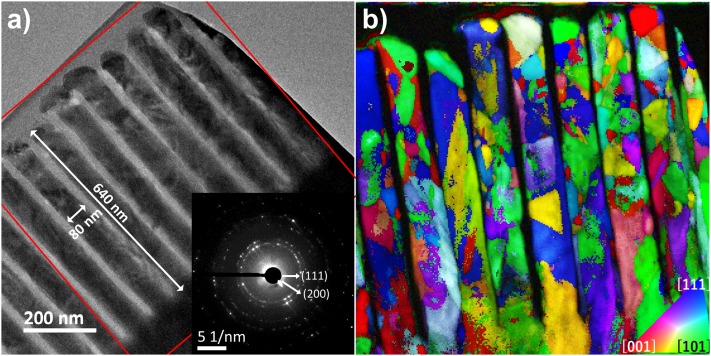
Microstructure analysis of the Ni nanowires: (a) TEM image of the Ni-NWs/AOO composite, its SAED pattern is shown in the inset; (b) Crystal orientation map, along the ‘Z’ direction, of the nanowire array with color code.

To visualize the magnetic distribution within a single row of polycristalline ferromagnetic nanowires, the in-plane component of magnetic induction among the composite was imaged using EH. For reference, Fig. 3a shows the hologram of a section of the array. The holograms were acquired under Lorentz conditions and analyzed using HoloWorks 5.0.7. Interference fringes were oriented longitudinally in the parallel direction of the nanowire with an average fringe spacing, σ = 12.5 nm in a field of view (FOV) of 2 μm^2^ and a fringe contrast of μ ≈ 35%. Fig. 3b presents the sample immediately after being prepared by FIB. For Fig 3c the retrieved phase of the sample was obtained after an external magnetic field of ∼18 kOe was applied to the sample in the microscope, lines contours and alterning color sequences are significantly more visible. Ni NWs segments have green and red colors (180° of orientation) while nickel on the top has a blue color rotated 90° with respect to NWs.

**FIG. 3.**
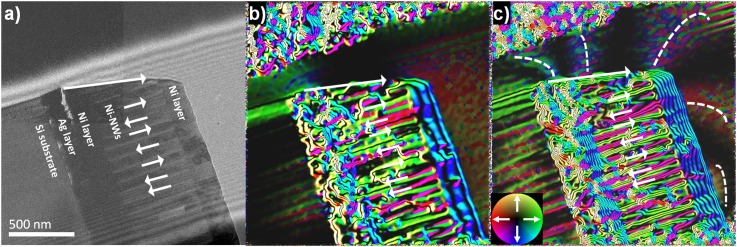
(a) Electron hologram of a single row of Ni nanowires. (b) Amplification of the magnetic/electrostatic contour lines representing phase variations through the plane of the sample. (c) Same as (b) after an external H (∼18 kOe) was applied perpendicular to the nanowires using the microscope objective lens coils, note the lines of the magnetic field outside the sample.

To obtain only the magnetic contribution in the array, the magnetic phase separation was carried out by recording holograms of the same FOV above the Curie Temperature of Nickel (358° C) using a heating TEM holder to collect reference holograms at 500°C. Once the two holograms are acquired a substraction is performed to eliminate the crystalline potential contribution from the unwrapped phase images.^25^ The magnetic induction image in Fig. 4a is formed from the phase gradient and weighted amplitude from the magnetic contribution phase image. Phase shift between opposite parallel directions (green/magenta) are around 5.4 rad, for a nanowire of 80nm of diameter this correspond to a magnetization of 5.5x10^5^ A/m.^26^ For demagnetization calculations, the geometry of the sample was replicated using the Gmsh three-dimensional finite element mesh generator software.^27^ The 30 nm region represents the contact zone between nanowires generated during growth. The 150 nm region is a nickel coating deposited over nanowires. Mesh element size was 8 nm in all cases. Simulations were performed with an exchange coupling constant A=7.6x10^-12^ J/m, saturation magnetization Ms=4.8x10^5^ A/m, and zero magnetocrystalline anisotropy.^28^ It is noted that the magnetic moments at both ends of the NW columns in Fig 4b start to reverse via a curling mode and that the magnetic moments in the middle of the NWs are aligned along the length axis as almost all grain orientations within the nanowire are equally favorable for spontaneous magnetization. In contrast, experimental work in systems like cobalt, with a strong easy axis along the [0001], favors the magnetic flux movement through that direction resulting on wavy rather than straight field lines.^26^ This result is consistent with the magnetic induction image showing interwire ferromagnetic (FM) ordering. The simulated demagnetization showed an alternation in the remaining field similar to that observed experimentally.

**FIG. 4.**
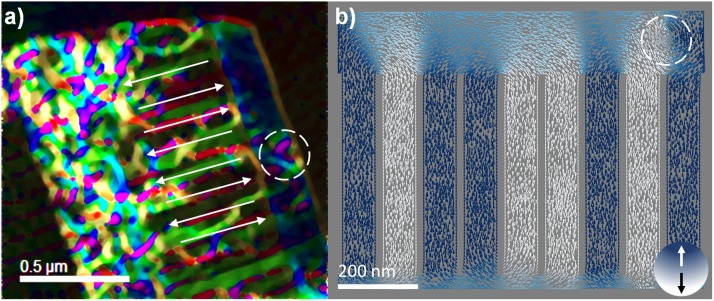
(a) Magnetic induction image reconstructed from subtracting holograms taken at 25°C and 500°C, showing FM interwire order (alternating green/magenta). (b) Nmag simulation where dimensions and distribution of the nanowires from image (a) were modeled. The row was saturated perpendicular to the nanowire axes and then the structure returned to a remnant state.

In summary, we have demonstrated a detailed investigation of the morphological, microstructure, and magnetic ordering of a Ni-NWs/AAO composite using in-situ heating and magnetization procedures. The microstructural investigation reveals the polycristallinity of the nanowires and their grain size and distribution. The electron holography directly visualizes the ferromagnetic ordering within the nickel columns, and both simulations and experimental images show a curling mode due to the adjacent nickel layers.
